# Design for Pride in the Workplace

**DOI:** 10.1186/s13612-016-0041-7

**Published:** 2016-07-04

**Authors:** Yichen Lu, Virpi Roto

**Affiliations:** Dept of Design, School of Arts, Design and Architecture, Aalto University, Hämeentie 135C, Helsinki, Finland

**Keywords:** Experience design, Pride experience, Design strategy, Self-focus, Other-focus, Short-term, Long-term

## Abstract

**Background:**

Pride is one of the most meaningful experiences in daily life. Many psychological studies emphasize self-oriented and event-based achievements as the main sources of pride, whereas work from organizational management considers pride as a collective attitude derived from other-focused activities and fostered by the sense of belongingness. Taking the interdisciplinary aspects of pride into account, this article addresses the challenge of how experience design can contribute to pride experience in the workplace.

**Methods:**

By cross-cutting theories from psychology and organizational management, this study introduces a framework of dynamic pride. The data includes 20 experience design cases that were specifically devoted to positive experiences in the context of the metal and engineering industry. 33 pride-related experience design goals were analyzed and categorized into the framework of pride.

**Results:**

This study introduces the social and temporal dimensions of pride experience at work. The pride-related experience design goals fall into four categories: self-focused short-term pride, self-focused long-term pride, other-focused short-term pride, and other-focused long-term pride. Accordingly, the extracted design strategies of these goals were mapped to each type of pride. Most of these design strategies were clustered in the categories of self-focused short-term pride and other-focused long-term pride.

**Conclusions:**

This study reveals the design strategies for dynamics of pride in the workplace varying from evoking self-achievement in individual interactions with tools to maintaining long-term motivation of self-competence development, and from highlighting one’s contribution in face-to-face collaborative work facilitated by interactive tools to fostering co-experience of organizational pride throughout social events.

## Background

People feel life holds more meaning when they are motivated by cherished goals, aware of self-improvement, involved in healthy interpersonal relationships, and loyal to their beliefs. In essence, these profound experiences of meaning make life worth living (e.g., Seligman and Csikszentmihalyi 2000). However, the contribution of subjective value experiences, especially those of happiness, has not gained adequate attention in empirical research until the emergence of positive psychology. This new branch of psychology shifted the research focus from pathology to optimal human function and flourishing (Seligman and Csikszentmihalyi 2000), and it addresses how to enable individuals and communities to thrive (Seligman 2011).

Positive psychology has promoted human flourishing as the ultimate goal of scientific research. It has been increasingly applied to other disciplines, such as education, policy, management, mental health, computer science, engineering, and design (Calvo and Peters 2014, p. 25). In the field of human–computer interaction, the “positive technology” approach was proposed to utilize interactive technologies for personal experience optimization (Riva et al. 2012). From the perspective of multidisciplinary efforts, Calvo and Peters (2014) refer to this area of design and development of technology for psychological wellbeing and human potential as “positive computing”.

Highly connected with technology and engineering, the discipline of design (e.g., industrial design, product design, and interaction design) has been inspired by the mindset of positive psychology: from preventing pain towards promoting happiness, from material sufficiency towards experiential value (Pohlmeyer 2012), from immediate response towards long-term impact, and from designing solutions towards designing possibilities (Desmet and Hassenzahl 2012; Jensen 2014). Human flourishing has essentially changed the traditional design process, exemplified by recent scholarly advice, such as “think experience before product” (Hassenzahl 2010) and “first decide what kind of experience to be aimed for and then design certain features to evoke the targeted experience” (Desmet and Schifferstein 2011). Design approaches, such as experience-based design (Bate and Robert 2007), experience-centered design (Wright and McCarthy 2010), experience-driven design (Desmet and Schifferstein 2011), positive design (Desmet and Pohlmeyer 2013), experience design (Hassenzahl et al. 2013) and design for profound experiences (Jensen 2014), prioritize quality experience goals over material-level requirements. One typical experience design case introduced by Hassenzahl (2010) is Philips wake-up light simulating sunrise and bird singing for natural wake-up experience in the morning. Combining an alarm clock and a bedside lamp, it guides user gently out of a deep sleep phase by progressively increasing in light intensity and volume of bird singing. Adding to this understanding of experience design, Lu and Roto (2014) defined an experience goal addressing in-depth meaning as the starting point and driver of design process. Functionality and usability requirements are submissive to ultimate experience goals.

To facilitate the designers’ adaptation to this mindset change, design researchers have started to translate knowledge from the field of psychology into design approaches. Hassenzahl et al. (2010) selected six out of 10 psychological needs (Sheldon et al. 2001) and suggested utilizing an “experience pattern” as a tool to distill the essence of an experience and transfer it to the targeted context. Desmet (2012) introduced a basic set of 25 positive emotion types and proposed six main sources of positive emotions in human product interactions. Desmet and Pohlmeyer (2013) created a framework suggesting three ingredients of design for human flourishing: pleasure, personal significance, and virtue. Calvo and Peters (2014) identified the determinant factors of wellbeing and provided the strategies to develop a certain factor as well as its evaluation methods. For the specific context of the industrial work environment, Lu and Roto (2015) borrowed the knowledge on the meaning of work (Rosso et al. 2010) and provided high-level design strategies for evoking meaningful experiences at work regarding work tool design, such as promoting competence for the perception of personal significance. These frameworks address positive experiences as the root of design, and open the door to design for wellbeing in general. However, these theories may arguably appear too essential, comprehensive, or concise for designers, and scarcely reach to the fine granularity required for design action. The nature of design practice remains highly context-dependent and the resulting design is required to be concrete and to manifest in details. Therefore, more explicit and practicable strategies for experience design are needed regarding a specific experience in a targeted context.

This study aims to investigate design strategies for human flourishing with a special focus on pride experience in the workplace, because the nature of pride is full of richness regarding meaningful experiences at work. According to the positive computing framework (Calvo and Peters 2014, p. 87), pride covers the dimension from *intra*-*personal* pride that is experienced within oneself (e.g., feelings of self-achievement) to *interpersonal* pride that is experienced from interaction between oneself and others (e.g., perception of respect from others). From the perspective of time, pride can be experienced as a moment-by-moment positive emotion evoked by unstable events, such as success in a challenging task. Also, pride can be considered as a cumulative experience based on a long-term rational attitude, such as loyalty towards one’s community. In contrast to the richness of pride, current design research studies provide limited sources of design for pride, which are mainly constrained by the mere scope of product design and the perspective of self-achievement and personal distinct possession (Desmet 2012).

Pride as a meaningful experience design goal in the workplace is worth investigating further. This study specifically strives to provide designers concrete design-for-pride strategies from two angles: the literature review on the multiple facets of pride and the empirical data on pride embodiment in design concepts. Accordingly, with a special focus on workplace, this study addresses two research questions: first, to identify what are the dimensions of pride that help designers to understand the design space for pride experience; second, to distinguish the strategies that designers have used so far in the design-for-pride cases. By synthesizing theoretical and empirical knowledge, this study proposes a multi-dimensional framework of design-mediated pride^1^ and the design strategies for evoking dynamics of pride in the workplace.

The remaining sections of this paper are structured accordingly: first, a theoretical framework of pride based on literature review is presented and the need of knowledge on design-mediated pride is identified; second, 20 cases that were designed for positive experience at work are analyzed based on self-focus to other-focus dimensions and short-term to long-term timespans; third, derived from these cases, the design strategies for evoking pride experience at work are proposed; finally, the insights from these design strategies are discussed.

## Literature Review

### Psychological Structure of Pride

Pride is a fundamental human emotion involving a complex self-evaluative process (Tracy and Robins 2004). Different from other “purely” basic emotions, such as the universally admired emotion of love or the universally reviled emotion of jealousy (Williams and DeSteno 2009), pride is comprised of two distinct facets. To this extent, Tangney (1990) referred to “alpha” pride as pride in self and “beta” pride as pride in behavior. Furthermore, Tracy and Robins (2004) distinguished between authentic pride and hubristic pride: authentic pride is evoked by accomplishment from successful behavior and positively related to genuine self-esteem and prosocial traits, whereas hubristic pride is more towards self-aggrandizement and positively related to narcissism (Tracy et al. 2014). Additionally, the two facets of pride differ from each other in cognitive antecedents (Tracy and Robins 2007b). Authentic pride is triggered more by unstable, specific, and controllable attributions, such as solid results due to hard work, whereas hubristic pride is more likely to occur from stable, global, and uncontrollable causes, such as feelings of superiority from “who I am” (Tracy and Robins 2007b). As this study focuses on designing for positive pride, hubristic pride is excluded in the scope of this paper.

#### Self-Focus Versus Other-Focus

Both self- and other-focused pride are sources of positive emotion (Desmet 2012); moreover, the categories of self and social have been identified as wellbeing factors (Calvo and Peters 2014). Self-focused pride emphasizes more on interaction within oneself and response to oneself whereas other-focused pride accentuates interpersonal interaction and the influence between self and others. Most studies emphasize pride as a self-conscious and performance-related experience triggered by self-efficacy (Tracy and Robins 2007a). Besides its elicitation through self-achievement, pride as a fundamental social emotion can also be “generated by appraisals when one is responsible for a socially valued outcome or for being a socially valued person” (Mascolo and Fischer 1995, p. 66). Moral accomplishment and prosocial actions are associated with the feeling of pride that may motivate and reinforce one’s socially valued conduct (Tangney et al. 2007), such as caregiving (Tracy and Robins 2007b), treating others well (Michie 2009), and positively responding to others’ emotions and needs (Leffel et al. 2008). Nakamura’s (2013) related work suggests that compared with self-oriented achievement, other-oriented prosocial action has an even stronger relationship to pride in both family life and work life. Therefore, no matter whether it is triggered by self-oriented task accomplishment or other-oriented altruistic activities, pride functions as both a “barometer” and “motivator” (McCullough et al. 2001) in assessing, regulating, and encouraging one’s behavior toward being “good, competent, and virtuous” (Haidt 2003, p. 860).

#### Short-Term Versus Long-Term

Pride derived from subjective histories of success may promote eagerness towards new anticipatory goals (Katzenbach 2003b). This promotion-related eagerness may energize and enhance performance (Higgins et al. 2001) and thus renew the experience of pride. As such, pride can transition from a temporary emotional experience towards a durable attitude of pride. According to the timespans of user experience (Roto et al. 2011), a new challenging goal may evoke an anticipatory pride for a person with a subjective history of success (Higgins et al. 2001); incremental progress in problem solving may elicit a momentary pride; when reflecting on an overcome challenge, a person may feel an episodic pride in the achievement. These performance-related types of pride are short-term, event-specific, and ascribed to internal attributes, such as ability or effort (Weiner 1985). Additionally, another kind of long-term and cognitive attitudinal pride exists in organizational studies, which does not rely on single events, but cumulative experience related to the overall evaluation of a target (Gouthier and Rhein 2011), such as being proud of one’s community.

### Pride Experience at Work

Pride is one of the most intense experiences in work life (Katzenbach 2003b), and work itself is a source of pride (Hodson 1998). Katzenbach (2003b) distinguishes institution-building pride which is based on largely intangible value and collective interest from self-serving pride which is driven by power and materialism.

Employees can take intrinsic pride in what they make, how they work, and whom they work with (Katzenbach 2003b). Experience of pride in achievement can be empathized by others in social interaction at work and thereby contribute to psychological empowerment and promote future successes (Froman 2010). Katzenbach (2003b) introduces a powerful “closed loop of energy” derived from pride: better performance contributes to business success, and recognized business success instills a strong feeling of pride, which fuels future better performance. This cycle can be repeatedly applied in organizational management.

Gouthier and Rhein (2011) discern two types of organizational pride: one is an emotional pride triggered by successful organizational events, and the other is a cognitive and durable attitude of pride oriented from the general perception of the organization and employees’ sense of belonging to the organization (Lea and Webley 1997). On this matter, the celebration of successful events, presence of a successful company history and culture, and successful advertising campaigns have been identified as activators of organizational pride (Gouthier and Rhein 2011).

**Fig. 1:**
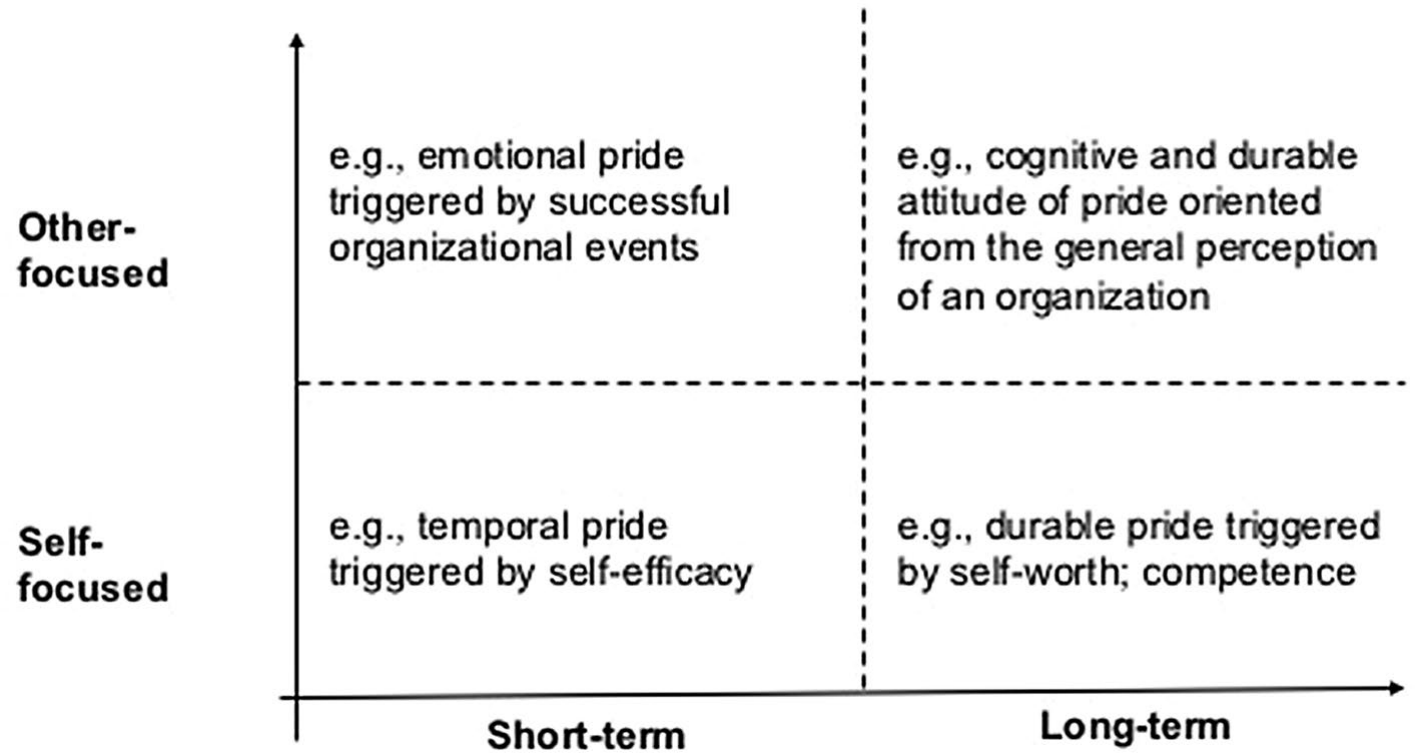
Social and temporal dimensions of pride experience at work

In summary, pride experience can be derived from self-focused achievement and other-focused interpersonal interaction. The richness of pride also lies in covering a timespan from a temporary emotion to a durable attitude. Pride can be intensively experienced in the workplace, and intrinsic pride can be evoked by organizational celebration and reputation. From the psychology and organizational management literature review, two dimensions of pride were identified relevant for design: social dimension from self-focused to other-focused and temporal dimension from short-term to long-term (Fig. 1).

### Design for Pride Experience

Recently, design researchers have started to explore how design can contribute to pride experience. Desmet (2012) introduced pride among the 25 positive emotions in the scope of human-product interactions: pride may be evoked by owning a unique product, being able to use a complex product, achieving something a product facilitates, or receiving positive feedback from others concerning the product one owns. Based on Desmet’s work, Yoon et al. (2013) developed a tool to facilitate emotional granularity in design and specified that pride may be triggered by “one’s praiseworthy behavior surpassing internal and external standard, and/or one recognizes that others appreciate it” (ibid., p.8). Compared with the two dimensions of pride (Fig. 1), pride as a collective experience in social interaction (Battarbee and Koskinen 2005) and the long-term cumulative experience has not yet been fully addressed in existing experience design theories.

One example of positive design for pride given by Desmet and Pohlmeyer (2013) is a designed social interactive activity in which patients with dementia can match record pieces in order to play music from their past on a turntable with others; thus, a feeling of pride may arise from task completion within the social interaction. Moreover, two cases of work tool design for meaningful experience at work (Lu and Roto 2015) indicate that employees may feel proud of mastering a tool and being part of an organization; meanwhile, customers may experience pride when they make a right decision on a tool purchase and present manufactured tools to their clients. These design examples suggest how designers shape the conditions that may evoke a pride experience in a specific context. These cases reveal different strategies of designing for dynamics of pride. On one hand, those theoretical sources of pride, such as achievement, competence, owning something special, and positive appraisal from self and others, are clearly embodied in these three cases. On the other hand, when referring to a pride experience in a specific context, designers seem to employ specific context-adapted design strategies, such as utilizing the positive relationship between personalized music and a patient with dementia, developing the employee’s perceptions of organizational reputation, and highlighting one’s contribution of a right decision to an organization.

Because the nature of design knowledge is highly applicable and practice-driven (Cross 2001), besides the theoretical perspective, it is meaningful to examine how different types of pride were designed for in the concrete cases. This study aims to help designers to discover profound and unique sources of pride experience in the specific context of the workplace. The design-for-pride strategies employed by the collected cases will be identified and analyzed against the theoretical dimensions of pride (Fig. 1).

## Methods

This study falls under research for design category of design research, since the main aim of this work is to improve design practice (Frayling 1993). In line with Zimmerman et al. (2010), this research for design activity yields to a framework and design recommendations that help designers in their work. Besides a literature review, the research data is also derived from empirical design studies. The specific design approach under study is experience design, in which experience goals are the key prerequisite for design activity and defined in the early stage of the design process. Experience goals drive the whole design process and evolve into the designed artifacts that may evoke the targeted experience (Lu and Roto 2015). Thus, this study proposes that the design strategies for shaping a certain experience can be distilled from the argumentation of the experience goal realization in the design concepts. According to the premise of experience design, any kind of design that best fulfills the experience goals can be the design outcome. In the workplace context, specifically, this may mean a tool, service, space, or even an event.

The authors of this paper studied the reports of 20 experience design cases that were conducted in collaboration with masters-level design students and seven companies in the metal and engineering industry from 2012 to 2015. The given design briefs from the different companies share the same high-level goal to design for meaningful experience at work in heavy industry. The differences among the 20 assignments lie in the design contexts varying from heavy machine operation (e.g., crane remote control, tugboat console, control room of automation system) to the peripheral touchpoints for different stakeholders involved in the industrial system (e.g., a mobile application for factory automation customers, a mobile sales application for ship components, a mobile crane monitoring application).

In the beginning of the design course, experience design approaches were taught to the students by at least one of the authors. Design teams were then comprised of two to three master students who worked full time on a two-month design assignment from one company. Each company assignment was tackled by one team of students. All the teams underwent the following design process: familiarizing oneself with the target context and users, defining a set of profound experience goals, deriving concepts from the determined goals, and finally evaluating design concepts against these goals with relevant stakeholders. Most teams produced two design concepts: one called ‘incremental’ to address the company’s current needs, and another called ‘radical’, which was supposed to radically improve the user experience and show what experience design could mean without limitations for the outcome. The students defined the experience goals based on different sources (Kaasinen et al. 2015), and they had the freedom to set up goals that would fit their case briefs. In each case, experience goals were formatted into a word or a short phrase for convenient communication among different stakeholders. There was no special rule with regard to how many experience goals the students should define, whereas the relations between a set of experience goals were expected for clarification. Involved throughout the whole design process, the company personnel were available for providing information and comments. At least one of the authors followed these cases by arranging weekly meetings with the design teams and reading their design diaries. The students were not given special guidance for designing for pride, as pride was not a presumed design goal in any of the assignments. Rather, the students were trained to identify multiple profound experience goals for the targeted workplace contexts. Only after a majority of cases ended up pride-related experience goals, the authors got interested in a deeper study of pride experience at work. Hence the analysis of the design cases was retrospective and the final reports of the student teams served as the primary data source for this part of the research.

### Four Design Case Examples

The design case description can provide better understanding of the experience design in the industrial workplace context. Due to the limited length of this article, this section will shortly describe four example cases that tackled pride from different perspectives.

**Fig. 2:**
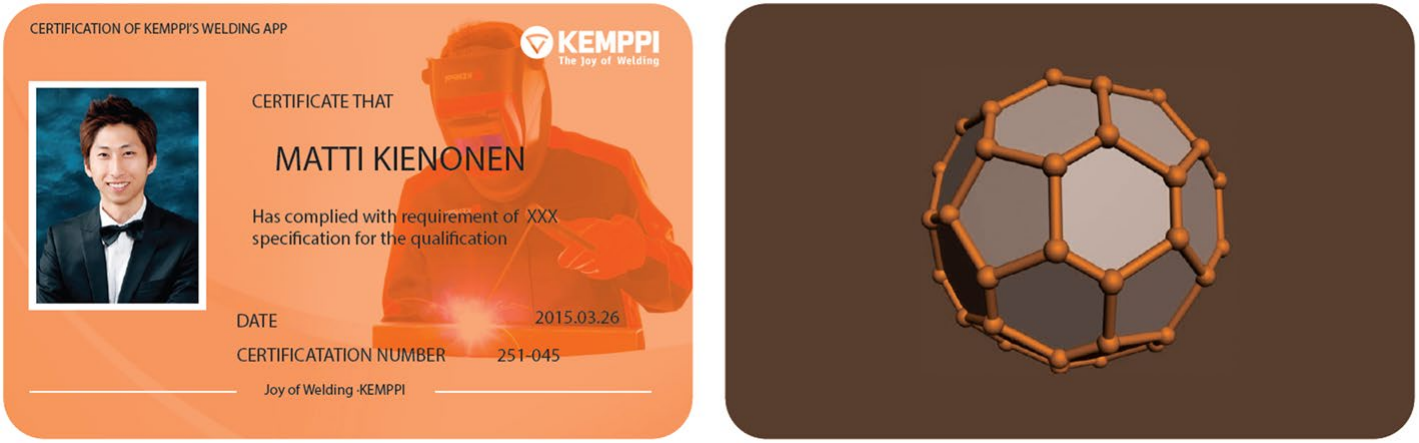
A physical certificate was designed to enhance pride after completing welding training

The customer for the first experience design case was Kemppi, a manufacturer of welding machines. The task was to design a mobile application with which people (e.g. welding students) could train their welding skills with welding without the actual welding equipment, but rather with the help of a welding simulator game. The design students drew three experience goals: Pleasure (the joy of welding), Self-motivation (willingness to practice constantly), and Pride (about their skills, the results, and the welding itself). The Pleasure goal was tackled by gamification features; Self-motivation by sharing and communicating the progress; and Pride by a physical certificate after completing all levels of training (Fig. 2). The incremental concept was a mobile application as suggested in the assignment, and the radical concept focused on the best possible pride experience by inviting selected application users to showcase their real-life welding skills in an event similar to an art exhibition.

**Fig. 3:**
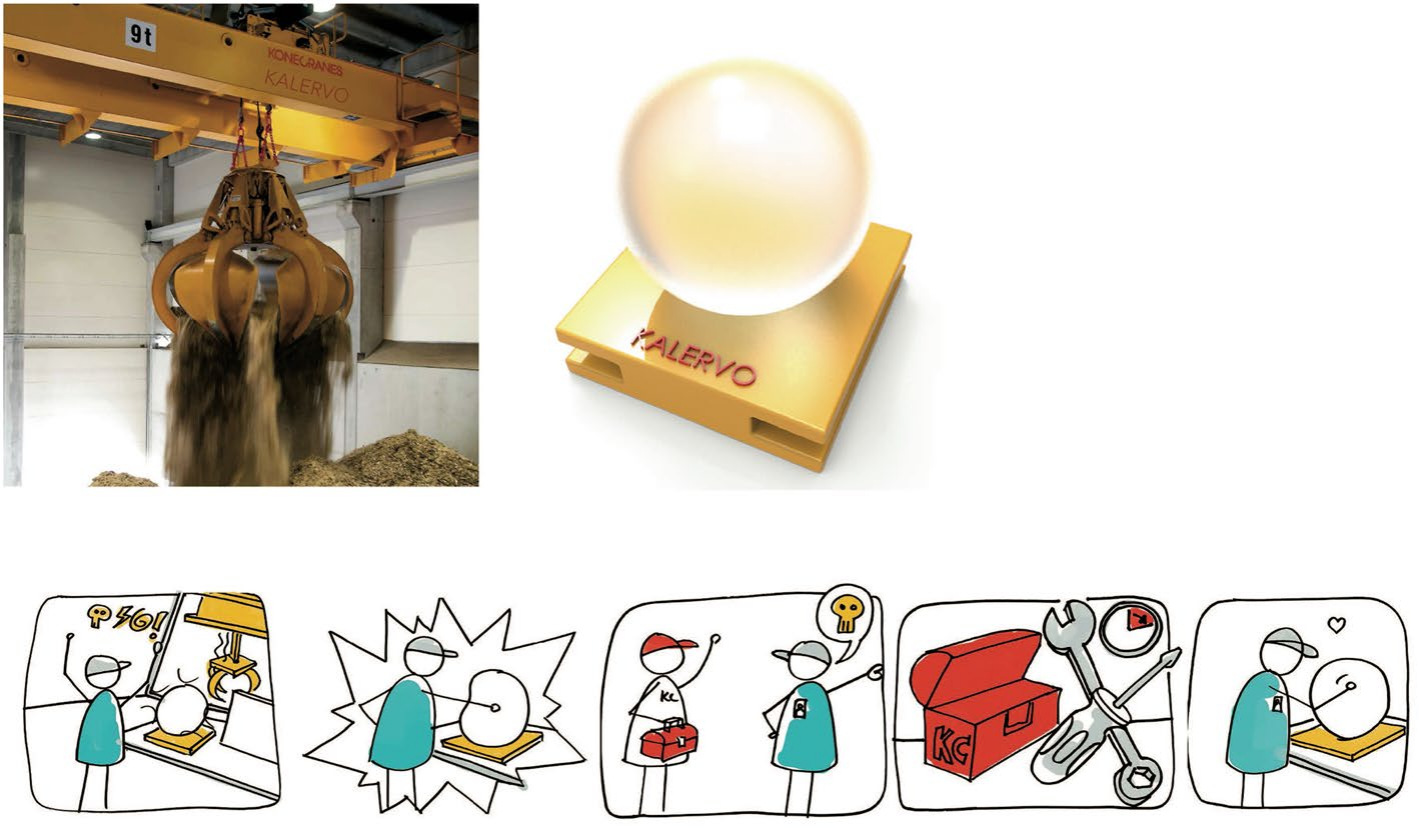
A light ball communicates the status of a crane to the crane operator, and the operator can send emotional messages to Konecranes service by interacting with the ball

Another kind of experience design case was completed for Konecranes, focusing on the maintenance services of cranes at waste-to-energy plants. After studying the employees at the plant, the students set goals for the crane maintenance service experience. Of the three goals, two were related to the pride experience: Worthiness (an affirmation of their importance as a customer) and Belongingness (a meaningful relationship with Konecranes employees). The radical concept is called Mood sphere, consisting of a light ball at the plant and an identity badge for each employee. Both the badge and the ball show the status of the crane and the related Konecranes service. The crane operators can interact with the ball to communicate their feelings about the crane and its service (Fig. 3).

**Fig. 4:**
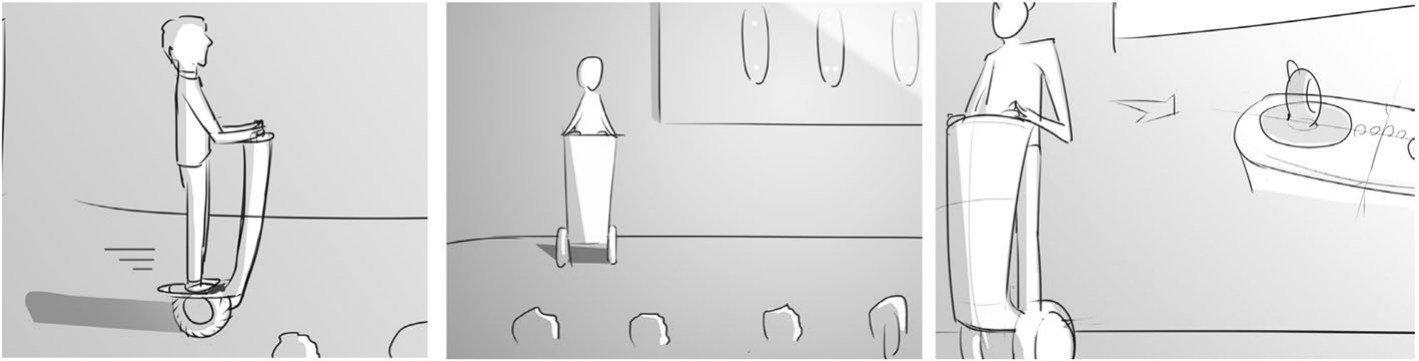
A salesperson presenting in front of an audience by driving a tugboat steering simulator

In the third case, students were asked to redesign a tugboat steering simulator for Rolls-Royce Marine. The objective was to create a quality experience both for the salesperson demonstrating the steering properties with different thruster options and for the customer wanting to see its functionality. The task for the students was focused on the physical design of the simulator, consisting of two big joystick devices and a display. The practical goal was to enable a portable system. The experience goals were derived with the salesperson’s experience in mind, since the salesperson is the primary user of the simulator. The students utilized the metaphor of Q from the James Bond movies and set three experience goals that all were related to the experience of pride: Sense of directing (directing the situation by suggesting possible solutions), Expertise (presenting oneself as a professional, technical expert), and Pride (proudly representing the company). The design strategy to fulfill the Sense of directing goal was to boost the feeling of control; Expertise goal by using impressive technology; and Pride by drawing attention with a novel way of presentation. The radical solution proposed by the students was a simulator vehicle, similar to a Segway, with the steering joysticks mounted on it (Fig. 4).

**Fig. 5:**
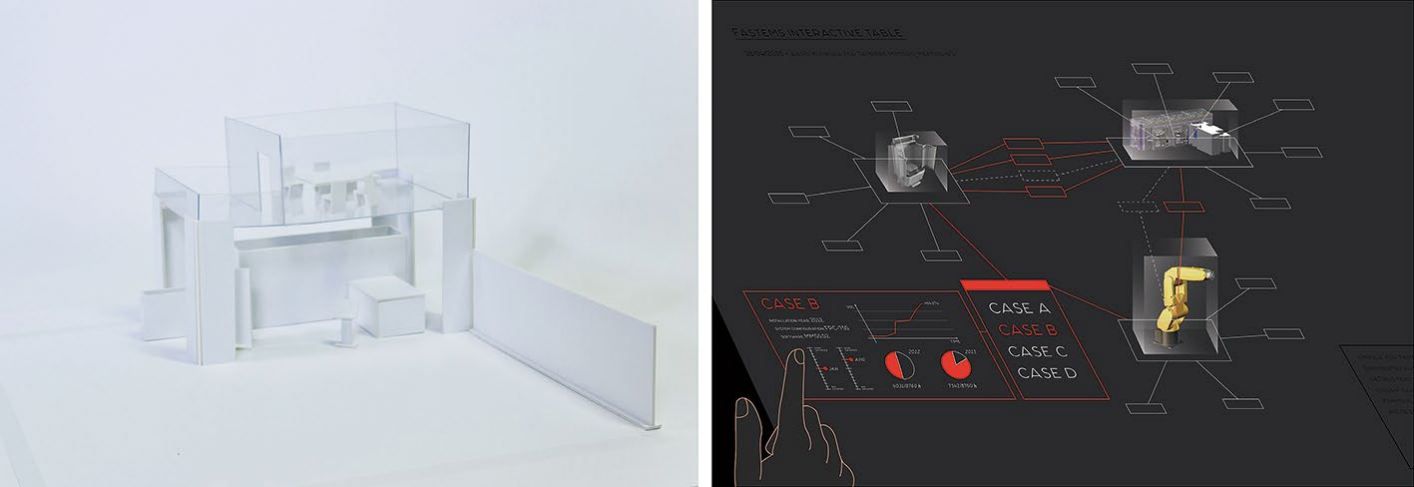
A physical space for visitors (*left*) and an interactive table for collaboration (*right*)

The last experience design task reported here was assigned by a factory automation company, Fastems, who wanted to extend their training center into a visitor space. In this case, the company already had defined company-wide experience goals (Roto et al. 2015) for experience development projects, from which the students derived four experience goals for this specific case. One of these was Participation for pride, which aims to foster a pride experience in customers by being able to influence the development activities at Fastems. In addition to the physical space design in the center of the factory activities, students proposed an interactive table as a collaboration platform• in the space (Fig. 5).

### Selecting Pride-Related Experience Goals

To decide whether a case is an endeavor for evoking a pride experience at work, the authors analyzed the final reports of all student teams, and examined the description of each experience goal setting and its infusion into a design concept. Compared to the existing guidance on designing for pride, this study adopted a broader and deeper understanding of pride based on the literature study. Besides the experience goals literally labeled with “pride”, those having the potential to elicit pride experience were also taken into account as pride-related experience goals and their relevance to pride is indicated in Table 1. The researchers interpreted the implication of these goals situated in the design context rather than their original meaning. For example, in the Kemppi case introduced above, pride as a long-term experience is based on a momentary pleasurable pride and episodic evaluative pride in self-motivation and thus Pleasure and Self-motivation are grouped into pride-related experience goals. On the other hand, if the experience goals and their embodiment in the final concepts both have little connection with pride, then these goals are excluded in this study.

**Table 1:** The experience goals with high relevance to pride

Pride-related experience goals	References
Sense of directing, expertise, excellence, competence, empowering, confidence, appreciation, usefulness, achievement, pleasure	Pride as a reaction to experiencing ‘mastery and achievement’ (e.g., Tracy and Robins 2007b)
Worthiness, self-esteem, self-actualization, self-motivation, being in a spot light, ambition	Pride highly relates to a person’s self-evaluation and self-respect (e.g., Tracy and Robins 2007a)
Engagement, connectivity, communication	Pride elicited by prosocial conduct or action benefiting others (e.g., Nakamura 2013)
Belongingness	Pride evoked by being part of an organization or organizational events (e.g., Gouthier and Rhein 2011)

In these 20 design cases, altogether 61 experience goals were identified and more than half (33) of them were pride-related. Meanwhile, only two cases out of 20 have little connection with pride: one stressed Discovery as an experience in taking an elevator; the other focused on a Trust experience evoked by the designed cover for an expensive ship component placed outside.

### Analyzing Pride-Related Experience Goals Against Two Dimensions of Pride

Each goal was then postulated to fall into the category either long-term or short-term experience. Two researchers (the authors) conducted the goal categorization independently, resulting to an inter-rater agreement of 87.88 %. Consensus was reached by discussion. These pride-related experience goals were also categorized along the self-focus or other-focus dimension. The inter-rater agreement in this case was 72.73 %, and the consensus was reached by discussion.

Combining the two categorizations, each selected experience goal was supposed to belong to one of these four groups: self-focused short-term pride, self-focused long-term pride, other-focused short-term pride, and other-focused long-term pride. Accordingly, the design strategies used by the students for different types of pride were compared within and across categories.

## Results

According to the social dimension, 17 out of 33 pride-related experience goals are self-focused, whereas the remaining 16 goals are other-focused. Along the temporal dimension, 15 out of 33 pride-related experience goals are short-term, and the rest are long-term. The experience goals for self-focus short-term pride and other-focus long-term pride both take one-third of all the pride-related experience goals whereas other-focus short-term pride was the least designed for.

**Fig. 6:**
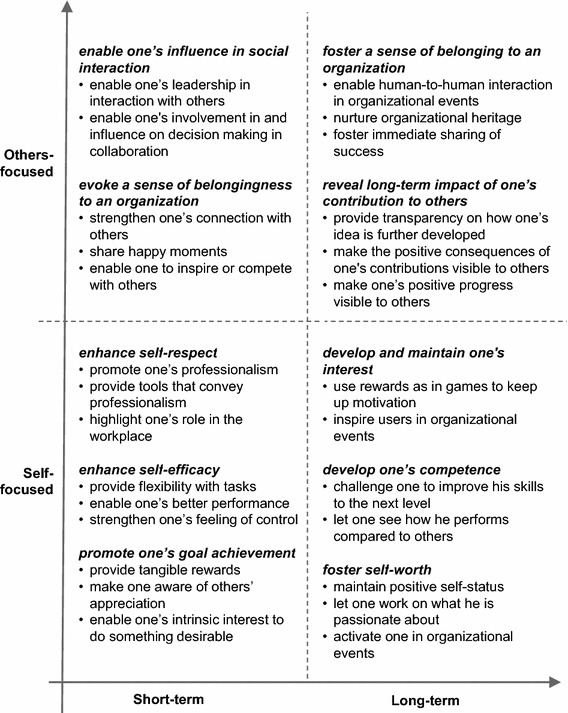
Design strategies sorted by two dimensions of pride

As shown in Fig. 6, different design strategies are summarized and grouped along the social and temporal dimensions of pride.

### Design for Self-Focus Short-Term Pride

10 out of the 33 pride-related experience goals are both self-focus and short-term. The design strategies are distilled into three aspects: enhancing self-respect, enhancing self-efficacy, and promoting one’s goal achievement.

#### Enhancing Self-Respect

Three experience goals indicate that enhancing the feeling of self-respect is a pathway to pride experience. Thus, providing users with a distinctive interactive tool or workspace and strengthening their unique expertise may increase self-awareness of users’ own professionalism, which leads to self-respect enhancement. For instance, in the Rolls-Royce Marine case introduced above, the salesperson drives in front of the customers a special and attractive vehicle that demonstrates the same technology in the tugboat steering. This unique way of presentation reflects the professional engineering knowledge of a tugboat salesperson.

#### Enhancing Self-Efficacy

Five experience goals suggest that evoking the feeling of control, improvement, and autonomy can enable momentary pride experience. For example, providing an overview of all the machines’ data may enhance a manager’s feeling of control; boosting performances with a new application may enhance a worker’s feeling of competence; providing flexible options of the control room layout may enhance an operator’s feeling of work autonomy.

#### Promoting One’s Goal Achievement

Two experience goals indicate goal achievement promotion may induce anticipatory pride. For example, rewarding a learner’s practice of welding with a tangible certificate and fueling one’s intrinsic interest in doing something desirable may be a pathway to pride promotion.

### Design for Self-Focus Long-Term Pride

Seven out of the 33 pride-related experience goals are designed for self-focused long-term pride. They stress three aspects: fostering self-worth, developing one’s competence, and developing and maintaining one’s interest.

#### Fostering a Sense of Self-Worth

Three goals indicate that activating one in organizational events, letting one work on what one is passionate about, and maintaining a positive self-status may foster self-worth, which would thus result in a durable self-focused pride. For example, in the case of Konecranes, the end users are often invited to a series of organizational events that may increase one’s awareness of their own value; making one passionate about one's work may result in self-actualization in a society; maintaining a decent status in a smooth transfer from work to retirement may increase one’s self-esteem.

#### Developing One’s Competence

Two goals indicate that challenging one to the next level of skills and letting one see how they perform compared to others may produce a durable and self-focused pride. In the case of Kemppi, the app provides gamification elements and makes the long-term skill improvement visible, which may in turn keep learners practicing through enjoyment of welding and competition with others.

#### Developing and Maintaining One’s Interest

Two goals from two cases indicate that motivating users with rewards as in games and keeping users’ inspirations in a series of organizational events may result in self-focus long-term pride. In the case of Kemppi, the app keeps an update of unlocked features to encourage learners onto the next practice level; a company can hold competitions, workshops, and seminars to maintain one’s interest and ambition and can thus accumulate one’s long-term pride.

### Design for Other-Focus Short-Term Pride

Five out of the 33 pride-related experience goals are both other-focus and short-term. They emphasize two aspects: enabling one’s influence in social interaction and evoking a sense of belongingness to an organization.

#### Enabling One’s Influence in Social Interaction

Two experience goals indicate that fueling one’s leadership in social interaction and revealing one’s influence on decision-making may result in other-focused short-term pride. In the Rolls–Royces case introduced above, the special vehicle may guide others’ attention and provide a salesperson with a sense of directing, thus facilitate the salesperson’s leadership in interacting with the audience. Moreover, enabling the customers’ involvement and hence developing awareness of their impact on decision-making may build pride in customers through social interaction.

#### Evoking a Sense of Belongingness to an Organization

Three experience goals indicate that facilitating a connection with others, sharing happy moments, and stimulating inspiration and appreciation from others may lead to the feeling of other-focus short-term pride. For example, the output of an interactive system at the entrance of the office may evoke employees’ belongingness to a company by worldwide company information dissemination and entertainment activity arrangement. Besides this, organizational events may have the similar impact on stakeholder engagement by stimulating their inspiration.

### Design for Other-focus Long-Term Pride

11 out of the 33 experience goals are designed for other-focus long-term pride. These goals focus on two main aspects: fostering a sense of belonging to an organization and revealing long-term impacts of one’s contribution to others.

#### Fostering a Sense of Belonging to an Organization

Six experience goals address the issue that enabling human-to-human interaction in organizational events, nurturing organizational heritage, and fostering immediate sharing of success may instill a sense of belonging to an organization and thus lead to other-focus long-term pride. For example, human-to-human interaction at work provides the feeling of camaraderie, the appreciation between each other, and the historical success of a company, which all contribute to collective pride towards an organization.

#### Revealing the Long-term Impact of One’s Contribution to Others

Five experience goals indicate that other-focused long-term pride can be produced by providing transparency on one's idea development, making the positive consequences of one's contributions visible to others, and making one’s positive progress visible to others. For example, the visualization of a process in which one’s work is further developed and implemented by others, and the positive confirmation of one’s critical decisions for an organization from a long-term perspective may increase one’s stable pride evoked by others.

## Discussion

Workplace as a social space provides a rich context for stimulation, development, and maintenance of the dynamics of pride. Design for pride in the workplace is a powerful, yet little utilized approach to keeping the closed loop of energy up for a successful business. By cross-cutting theories from psychology and organizational management, this study first introduces the social and temporal dimensions of pride. Based on the theoretical framework of pride, the empirical data from 20 experience design cases reveals contextualized and concrete design strategies for the dynamics of pride experience at work. The design implication drawn from these design strategies is given as follows.

The design strategies for self-focused short-term pride is in line with the sources of pride in human-production interaction (Desmet 2012): self-efficacy enhancement is related to “using the tool induces pride of task performance”; self-respect enhancements matches with “owning the tool induces pride of one’s expertise”; and goal achievement promotion fits with “the tool enables results that induces pride of one’s task performance”. To evoke such pride much depends on a well-designed momentary interaction between tool and user, for example, by measuring and visualizing incremental performance improvement in time.

When moving to a self-focus long-term pride, the design strategies adapt to the long-term effect, such as motivation maintenance, competence development and fostering self-worth. The key to designing for this type of pride is to explore and personalize the individual intrinsic meaning and to hold it longer, which is in line with positive design for personal significance, i.e., not focusing on the momentary effect, but on one’s personal goals and aspirations (Desmet and Pohlmeyer 2013).

Besides the facilitation of work performance enhancement, interactive tools can also be designed for other-focused short-term pride to assist users’ leadership or involvement in momentary social interaction, such as sharing one’s ideas by instant prototyping for collaborative discussion. Additionally, seeing others’ responses to one’s contribution may happen in organizational events, such as competitions, workshops, and seminars. These kinds of events may strengthen one’s connection with others, trigger social interaction, and stimulate each other’s creativity, in which co-experience of pride can be evoked within an organization.

Other-focused long-term pride is highly related to entrepreneurial spirit and loyalty. Company leaders aim to instill such pride into their employees and customers. The implications from organizational management can enter into design strategy by fostering a sense of belonging to an organization through activities (Gouthier and Rhein 2011). Belongingness is an other-oriented communion, which is about sharing common social identity and strengthening interpersonal connectedness (Rosso et al. 2010). Thus, sharing a positive identity is an important source of the other-focused pride, especially in organizational work contexts. Meanwhile, the visualization of progress and metrics can enable employees to track their own and others’ work (Katzenbach 2003b), and thereby reveal the long-term impact of one’s contribution to others, which is also a key design strategy for other-focused pride.

These four types of pride apparently connect to each other and work together. For example, one’s pride derived from the pleasure of interaction with a work tool may bring out both enjoyment and self-enhancement at work. The pleasurable pride may serve as an intrinsic motivator for long-term good work performance. The visualization of one’s positive work results and progress may evoke others’ appreciation. In the long run, the collective impacts of everyone’s incremental contribution accumulate and sustain organizational success and thereby lead to a durable pride towards one’s organization.

In conclusion, this paper identifies two main trends in designing for pride experience in the workplace: designers can uplift their vision from self-focused and achievement-oriented interaction with a tool towards fostering engagement-oriented interaction with people, and from event-based emotional pride in momentary interaction towards a long-term organizational attitude of pride. Meanwhile, the role of a tool becomes that of an interactive facilitator for co-experience of pride in activities rather than the passive means of task completion.

This study opens the discussion that experience design researchers need to study external knowledge in order to broaden the understanding of targeted experience, associate multiple dimensions of such experience with the design context, and then transfer the constructed knowledge into experience design strategy for concept generation.

## Limitation and Future Development

This study is a primary exploration on how to design for a specific positive experience in a targeted context. From the study approach perspective, the design strategies distilled from the triangulation between theories and empirical data were born with considerable validity which lies in the traceable evidences from available knowledge and concrete design cases. On the other hand, however, the predefined four-type-pride framework might limit the variety of the strategies, because there might be valuable special strategies that could be excluded by the scope of this framework. This limitation reflects that it could be a danger if designers overly rely on the available strategies and thus they might be restricted by the structured framework. In this sense, these identified patterns should serve more for understanding, inspiring and framing new themes, instead of fixing designers’ mindset.

From the data perspective, it is based on the collection of 20 student design cases in one design department from 2012 to 2015, which determines the specificity and limitation of this study. The interference between different design teams and the bias influenced by the earlier cases may be hardly avoided in the design process, which may lead to limitations of experience goal setting and idea generation. Although the fresh eyes of students may generate novel ideas and explore new possibilities, their limited breadth of mature design experience for the workplace may also result in some shallow design concepts. Moreover, a 2-month project can hardly include a long-term evaluation of design results.

From the finding validity perspective, the pride experience design strategies were presented as backup tools in the latest course but without guiding the students to use them. Compared with previous cases, pride was defined more towards durable pride and organizational pride in the recent cases. More importantly, inspired by these cases, the companies have gradually changed their mindset from focusing on usability towards prioritizing experience, and have recognized pride can be designed from not only interaction quality in task but also organizational impact. This impact was manifested in the industrial seminar when two companies presented and referred the student cases as their new path to experience design innovation. At least one design-for-pride case was implemented entirely from the students’ concepts, and got positive feedback from the company’s clients.

Future studies will focus on at least three directions. First, it is worth adding case diversity to the data collection: cases from different programs in different research institutes or companies are needed for both qualitative and quantitative studies to assess whether the two dimensions of design for pride can be applied to a large number of design cases. Second, there is a definite need to trace the real implementation of certain strategies in the client companies to identify the long-term impact on both workers’ subjective wellbeing and business development. Third, it is also meaningful to observe designing for pride in other domains and to identify what kind of strategies are common patterns and to what degree the differences of contexts lead to the specificity of design strategies.

## Footnotes

1 Thanks to the anonymous Reviewer 1.
